# Stability and bifurcation analysis of super- and sub-harmonic responses in a torsional system with piecewise-type nonlinearities

**DOI:** 10.1038/s41598-021-03088-z

**Published:** 2021-12-08

**Authors:** Jong-Yun Yoon, Byeongil Kim

**Affiliations:** 1 Department of Mechatronics Engineering, Incheon National University, Incheon, 22012 Republic of Korea; 2 School of Mechanical Engineering, Yeungnam University, Gyeongsan, 38541 Republic of Korea

**Keywords:** Engineering, Mechanical engineering

## Abstract

The nonlinear dynamic behaviors induced by piecewise-type nonlinearities generally reflect super- and sub-harmonic responses. Various inferences can be drawn from the stability conditions observed in nonlinear dynamic behaviors, especially when they are projected in physical motions. This study aimed to investigate nonlinear dynamic characteristics with respect to variational stability conditions. To this end, the harmonic balance method was first implemented by employing Hill’s method, and the time histories under stable and unstable conditions were examined using a numerical simulation. Second, the super- and sub-harmonic responses were investigated according to frequency upsweeping based on the arc-length continuation method. While the stability conditions vary along the arc length, the bifurcation phenomena also show various characteristics depending on their stable or unstable status. Thus, the study findings indicate that, to determine the various stability conditions along the direction of the arc length, it is fairly reasonable to determine nonlinear dynamic behaviors such as period-doubling, period-doubling cascade, and quasi-periodic (or chaotic) responses. Overall, this study suggests analytical and numerical methods to understand the super- and sub-harmonic responses by comparing the arc length of solutions with the variational stability conditions.

## Introduction

In the investigation of nonlinear dynamic responses, the stability conditions can contain important information, which may induce dynamic variations ranging from simple periodic responses to complex dynamic behaviors, including period-doubling, quasi-periodic, and chaotic motions^1–15^. To examine the complex dynamic behaviors, this study employs the harmonic balance method (HBM) using Hill’s method, which reveals the stability conditions of the system^16,17^. In addition, a numerical simulation (NS) was conducted to examine the system responses in the phase plane diagrams and Poincare maps. The comparisons of nonlinear motions based on the HBM with phase diagrams and Poincare maps focused on the variational stability regimes allow the physical dynamic behaviors to be more comprehensible.

With regard to the methods employed in this study, various works have been conducted previously. For example, Peng et al. suggested nonlinear output frequency response functions (NOFRFs) as an HBM using the Duffing oscillator^18^. To implement NOFRFs in strong nonlinear equations, they employed the Volterra series to extend the classic FRF to the nonlinear case. Chen et al. used the incremental harmonic (IHB) method to investigate the limit cycle oscillation of a two-dimensional airfoil with parameter variability in an incompressible flow^19^. Here, the strong nonlinear cubic stiffness subject to either non-probability but bounded uncertainty or bounded stochastic parameters was estimated using the IHB method. Duan et al. suggested an excitation perturbation method to investigate sub-harmonic resonance^20^. To capture the sub-harmonic effects, the authors modified the input conditions. For example, the relevant sub-harmonic input terms were artificially included, which triggered the corresponding sub-harmonic responses. Additionally, various approaches have been developed by employing a multiterm HBM to examine the nonlinear frequency responses in a torsional system with clearance-type nonlinearity^21–27^.

To advance the existing pool of knowledge based on the HBM and its relevant techniques, this study suggests a method to investigate the nonlinear dynamic characteristics that occur in the super- and sub-harmonic regimes, which mostly focus on the analysis of the stability conditions along the arc-length direction under the frequency upsweeping condition. Thus, the specific objectives of this study are as follows: (1) to investigate the dynamic characteristics in the super- and sub-harmonic areas with variational stability conditions using the HBM. The stability variations along the direction of the arc length can present the close relationships of the system response to various nonlinear motions such as period-doubling, period-doubling cascade, and chaotic which was not examined in the prior studies^17,28^; (2) to analyze the nonlinear dynamic responses by comparing the bifurcations with the stability conditions. To understand the complex dynamic conditions, FFT results, phase diagrams, and Poincare maps are also compared with the bifurcations. To resolve these issues, this study focuses on a specific multi-staged clutch damper model in a torsional system with 1DOF^17,28^, which will lead to understanding the vibration issues caused in the vehicle driveline system and give the better guideline to reduce the annoying noise and vibrational problems.

## Problem formulation and its basic formulation

### System modeling and piecewise-type nonlinearities

**Figure 1:**
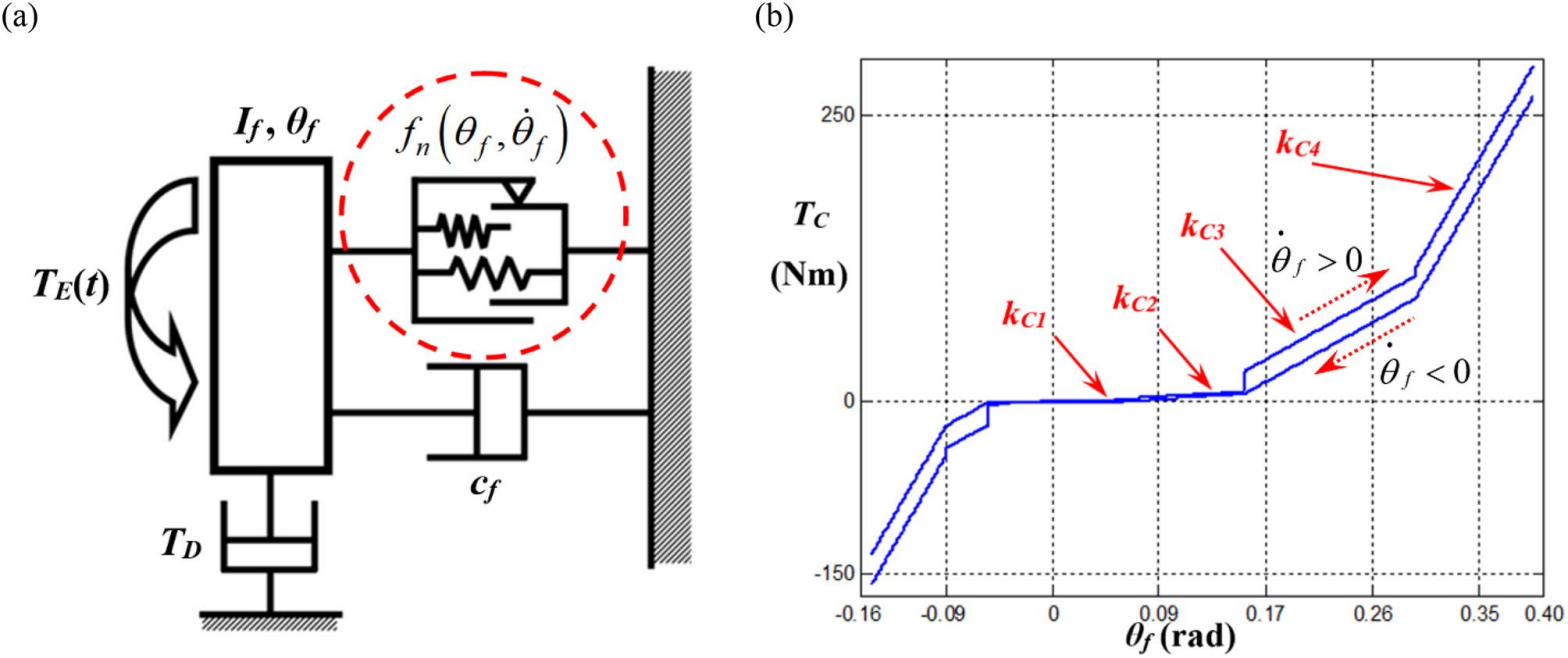
Torsional system with 1DOF affected by piecewise-type nonlinearities: (**a**) a nonlinear torsional system based on physical clutch dampers; (**b**) an asymmetric torque *T*_*C*_(*δ*_*1*_) profile induced by multi-staged clutch dampers^17,28^.

Figure 1a depicts a nonlinear torsional system with piecewise-type nonlinearities. Here, the nonlinear system with 1DOF is a part of a driveline focused on the flywheel and multi-staged clutch dampers based on prior studies^17,28^. The effective nonlinearities are based on asymmetric torsional springs with dry friction materials. To analyze the nonlinear force ${f}_{n}\left({\theta }_{f},{\dot{\theta }}_{f}\right)$ (or ${T}_{C}$) induced by the piecewise-type nonlinearities, mathematical formulations can be derived by considering the hysteresis effects as illustrated with the red dotted arrows in Fig. 1b^17,28^.

Here,$\theta_{f}$ and $\dot{\theta }_{f}$ represent the angular displacement and velocity of the flywheel (subscript $f$), respectively, as indicated in Fig. 1a. First, the clutch torque $T_{S} \left( {\theta_{f} } \right)$ induced by the stiffness with a smoothening factor $\sigma_{C}$ of $1 \times 10^{3}$ is derived as follows:

1a $$ T_{S} \left( {\theta_{f} } \right) = k_{C1} \theta_{f} + \frac{1}{2}\mathop \sum \limits_{i = 2}^{N} \left( {k_{C\left( i \right)} - k_{{C\left( {i - 1} \right)}} } \right)\left( {T_{{sp\left( {i - 1} \right)}} - T_{{sn\left( {i - 1} \right)}} } \right), $$

1b $$ T_{sp\left( i \right)} = \left( {\theta_{f} - \phi_{p\left( i \right)} } \right)\left[ {tanh\left\{ {\sigma_{C} \left( {\theta_{f} - \phi_{p\left( i \right)} } \right)} \right\} + 1} \right], $$

1c $$ T_{sn\left( i \right)} = \left( {\theta_{f} + \phi_{n\left( i \right)} } \right)\left[ {tanh\left\{ {\sigma_{C} \left( {\theta_{f} + \phi_{n\left( i \right)} } \right)} \right\} - 1} \right]. $$

Here, *k*_*C*(*N*)_ (or *k*_*C*(*i*)_) is the *N*th (or *i*th) stage of the clutch stiffness (with subscript *N* or *i*), *T*_*sp*(*i*)_ (or *T*_*sn*(*i*)_) denotes the positive (or negative) side of the clutch torque induced by the stiffness at the *i*th stage (with subscript *p* or *n*), and $\phi_{p\left( i \right)}$ (or − $\phi_{n\left( i \right)}$) represents the *i*th transition angle of the positive (or negative) side. Furthermore, the clutch torque *T*_*H*_ induced by hysteresis is considered with a smoothening factor $\sigma_{H}$ of 0.1.

2a $$ T_{H} \left( {\theta_{f} ,\dot{\theta }_{f} } \right) = \frac{{H_{\left( N \right)} }}{2}tanh\left( {\sigma_{H} \dot{\theta }_{f} } \right) + \mathop \sum \limits_{i = 2}^{N} \left( {\frac{{H_{\left( i \right)} }}{4} - \frac{{H_{{\left( {i - 1} \right)}} }}{4}} \right)\left[ {T_{{Hp\left( {i - 1} \right)}} + T_{{Hn\left( {i - 1} \right)}} } \right], $$

2b $$ T_{Hp\left( i \right)} = tanh\left\{ {\sigma_{C} \left( {\theta_{f} - \phi_{p\left( i \right)} } \right)} \right\}\left[ {1 + tanh\left( {\sigma_{H} \dot{\theta }_{f} } \right)} \right], $$

2c $$ T_{Hn\left( i \right)} = tanh\left\{ {\sigma_{C} \left( {\theta_{f} + \phi_{n\left( i \right)} } \right)} \right\}\left[ {1 - tanh\left( {\sigma_{H} \dot{\theta }_{f} } \right)} \right]. $$

Here, *H*_*N*_ (or *H*_(*i*)_) is *the N*^*th*^ (or *i*th) stage of hysteresis (with subscript *N* or *i*), and *T*_*Hp*(*i*)_ (or *T*_*Hn*(*i*)_) is the positive (or negative) side of the clutch torque induced by hysteresis at the *i*th stage (with subscript *p* or *n*). In addition to the torque calculated using Eqs. (1) and (2), the preload *T*_*Pr*_ must be considered as a function of $\theta_{1pr}$.

3a,b $$ T_{SPr} \left( {\theta_{1pr} } \right) = \frac{1}{2}T_{Pr1} \left[ {tanh\left( {\sigma_{C} \theta_{1pr} } \right) + 1} \right] + \frac{1}{2}T_{Pr2} \left[ { - tanh\left( {\sigma_{C} \theta_{1pr} } \right) + 1} \right], \theta_{1pr} = \theta_{f} - \phi_{Pr} . $$

Here, *T*_*SPr*_ is the total clutch torque induced by the preload, *T*_*Pr1*_ (or *T*_*Pr2*_) denotes the positive (or negative) torque induced by the preload, and $\phi_{Pr}$ represents the angle located at the preload. Overall, the total clutch torque is estimated by the summation of $T_{S} \left( {\theta_{f} } \right)$, $T_{H} \left( {\theta_{f} ,\dot{\theta }_{f} } \right)$, and $T_{SPr} \left( {\theta_{1pr} } \right)$ from Eqs. (1)–(3) as follows:

4 $$ f_{n} \left( {\theta_{f} ,\dot{\theta }_{f} } \right) = T_{C} \left( {\theta_{1pr} ,\dot{\theta }_{1pr} } \right) = T_{S} \left( {\theta_{1pr} } \right) + T_{H} \left( {\theta_{1pr} ,\dot{\theta }_{1pr} } \right) + T_{SPr} \left( {\theta_{1pr} } \right). $$

In addition, this study employed relatively higher value for $\sigma_{C}$ and lower one for $\sigma_{H}$ based on the prior studies^17,28^ since the linear torsional springs changes suddenly and the hysteresis effects are reflected smoothly^15,17,28^. The profiles of the clutch torque $f_{n} \left( {\theta_{f} ,\dot{\theta }_{f} } \right)$ (or *T*_*C*_) are listed in Table 1^17,28^.

**Table 1:** Properties of the physical multi-staged clutch damper.

Property	Stage	Value
Torsional stiffness, *k*_*Ci*_ (linearized in a piecewise manner) (Nm/rad)	1	10.1
	2	61.8
	3	595.8
	4	1838.0
Hysteresis, *H*_*i*_ (Nm)	1	0.98
	2	1.96
	3	19.6
	4	26.5
Transition angle at positive side (*θ*_*f*_ > *0*), ${\phi }_{pi}$ (rad)	1	0.05
	2	0.16
	3	0.30
	4	0.39
Transition angle at negative side (*θ*_*f*_ < *0*), ${\phi }_{ni}$ (rad)	1	− 0.04
	2	− 0.05
	3	− 0.09
	4	− 0.15

Figure 1b illustrates the 1st to 4th stages of the stiffness and hysteresis areas. To investigate the dynamic characteristics, the parameters employed for the torsional system shown in Fig. 1a are as follows^7^: inertia of flywheel, *I*_*f*_ = 1.38 × 10^−1^ kg·m^2^; viscous damping, *c*_*f*_ = 1.59 N m s/rad. Based on the single-degree-of-freedom system shown in Fig. 1a, the equation of motion with the nonlinear function $f_{n} \left( {\theta_{f} ,\dot{\theta }_{f} } \right)$ is derived as follows:

5 $$ I_{f} \ddot{\theta }_{f} \left( t \right) + c\dot{\theta }_{f} \left( t \right) + f_{n} \left( {\theta_{f} ,\dot{\theta }_{f} } \right) = T_{E} \left( t \right) - T_{D} . $$

Here, *T*_*E*_(*t*) and *T*_*D*_ are the sinusoidal input and drag torques, respectively. In general, the input torque is given by the Fourier coefficients based on the measured data as follows:

6 $$ T_{E} \left( t \right) = T_{m} + \mathop \sum \limits_{i = 1}^{{N_{max} \sum }} T_{pi} cos\left( {i\omega_{p} t + \varphi_{pi} } \right). $$

Here, *T*_*m*_ and *T*_*pi*_ are the mean and alternating parts of the input torque, respectively; $\omega_{p}$ and $\varphi_{pi}$ denote the excitation frequency and phase angle, respectively; and *N*_max_ represents the maximum number of harmonics correlated with the harmonic index of the HBM. The input torque profiles employed are listed in Table 2. In this study, the drag torque is assumed as *T*_*D*_ = *T*_*m*_ under steady-state conditions.

**Table 2:** Input torque profiles assuming the WOT condition on the vehicle.

Torque component		
Magnitude (Nm)		Phase (rad)
*T*_*M*_	*T*_*p1*_	
168.9	251.5	− 1.93

### Development of HBM from the governing equation

The Galerkin scheme in Eq. (5) is expressed as follows^17^:

7 $$ - \omega^{2} m\underline{{{\underline{ \mathbf{H} }}}} \underline{{{\underline{ \mathbf{P} }}}}^{^{\prime\prime}} \underline{{\theta_{c} }} + \omega c\underline{{{\underline{\mathbf {H} }}}} \underline{{{\underline{\mathbf {P} }}}}^{{\prime}} \underline{{\theta_{c} }} + \underline{{{\mathbf{f}}_{{\mathbf{n}}} }} \left( {\underline{{{{\varvec{\uptheta}}}_{{\mathbf{f}}} }} ,\underline{{{\dot{\mathbf{\theta }}}_{{\mathbf{f}}} }} } \right) - \underline{{{\mathbf{F}}_{{\mathbf{E}}} }} \left( t \right) = \underline {0} . $$

Its corresponding formulae are defined below.

8a,b $$ \underline{{{{\varvec{\uptheta}}}_{{\mathbf{f}}} }} \left( t \right) = \underline{{{\underline{\mathbf {H} }}}} \underline{{{{\varvec{\uptheta}}}_{{\mathbf{c}}} }} ,\quad \underline{{{{\varvec{\uptheta}}}_{{\mathbf{f}}} }} \left( t \right) = \left[ {\begin{array}{*{20}c} {\theta_{f} \left( {t_{0} } \right)} & {\theta_{f} \left( {t_{1} } \right)} & \cdots & {\theta_{f} \left( {t_{m - 2} } \right)} & {\theta_{f} \left( {t_{m - 1} } \right)} \\ \end{array} } \right]^{T} , $$

8c $$ \underline{{{{\varvec{\uptheta}}}_{{\mathbf{c}}} }} = \left[ {\begin{array}{*{20}c} {\theta_{m} } & {\theta_{a\left( 1 \right)} } & {\theta_{b\left( 1 \right)} } & \cdots & {\theta_{a\left( k \right)} } & {\theta_{b\left( k \right)} } & \cdots & {\theta_{{a\left( {\eta N_{\max } } \right)}} } & {\theta_{{b\left( {\eta N_{\max } } \right)}} } \\ \end{array} } \right]^{{\text{T}}} , $$

8d $$ {\underline{\mathbf {H} }} = \left[ {\begin{array}{*{20}c} 1 & \cdots & {cos\left( {k\psi_{0} } \right)} & {sin\left( {k\psi_{0} } \right)} & \cdots \\ 1 & \cdots & {cos\left( {k\psi_{1} } \right)} & {sin\left( {k\psi_{1} } \right)} & \cdots \\ & \ddots & & & \ddots \\ 1 & \cdots & {cos\left( {k\psi_{N - 2} } \right)} & {sin\left( {k\psi_{N - 2} } \right)} & \cdots \\ 1 & \cdots & {cos\left( {k\psi_{N - 1} } \right)} & {sin\left( {k\psi_{N - 1} } \right)} & \cdots \\ \end{array} } \right],\;\underline{{{\underline{\mathbf {H} }}}}^{{\prime}} = \omega \underline{{{\underline{\mathbf {H} }}}} \underline{{{\underline{\mathbf {P} }}}}^{{\prime}} ,\;\underline{{{\underline{\mathbf {H} }}}}^{{{{\prime\prime}}}} = - \omega^{2} \underline{{{\underline{\mathbf {H} }}}} \underline{{{\underline{\mathbf {P} }}}}^{{\prime \prime }} , $$

8e,f $$ \underline{{{\underline{\mathbf {P} }}}}^{{\prime}} = \left[ {\begin{array}{*{20}c} 0 & & & \\ & \ddots & & \\ & & {\left[ {\begin{array}{*{20}c} 0 & k \\ { - k} & 0 \\ \end{array} } \right]} & \\ & & & \ddots \\ \end{array} } \right],\quad \underline{{{\underline{\mathbf {P} }}}}^{{{\prime\prime}}} = \left[ {\begin{array}{*{20}c} 0 & & & \\ & \ddots & & \\ & & {\left[ {\begin{array}{*{20}c} {k^{2} } & 0 \\ 0 & {k^{2} } \\ \end{array} } \right]} & \\ & & & \ddots \\ \end{array} } \right]. $$

Likewise, its nonlinear and input functions are defined as follows.

9a,b $$ \underline{{{\mathbf{f}}_{{\mathbf{n}}} }} \left( {\underline{{{{\varvec{\uptheta}}}_{{\mathbf{f}}} }} ,\underline{{{\dot{\mathbf{\theta }}}_{{\mathbf{f}}} }} } \right) = \underline{{{\underline{\mathbf {H} }}}} \underline{{{\mathbf{f}}_{{{\mathbf{nc}}}} }} ,\quad \underline{{{\mathbf{F}}_{{\mathbf{E}}} }} \left( t \right) = \underline{{{\underline{\mathbf {H} }}}} \underline{{{\mathbf{F}}_{{{\mathbf{Ec}}}} }} , $$

9c $$ \underline{{{\mathbf{f}}_{{{\mathbf{nc}}}} }} = \left[ {\begin{array}{*{20}c} {f_{m} } & {f_{a\left( 1 \right)} } & {f_{b\left( 1 \right)} } & \cdots & {f_{a\left( k \right)} } & {f_{b\left( k \right)} } & \cdots & {f_{{a\left( {\eta N_{\max } } \right)}} } & {f_{{b\left( {\eta N_{\max } } \right)}} } \\ \end{array} } \right]^{{\text{T}}} , $$

9d $$ \underline{{{\mathbf{F}}_{{{\mathbf{Ec}}}} }} = \left[ {\begin{array}{*{20}c} {F_{m} } & {F_{a\left( 1 \right)} } & {F_{b\left( 1 \right)} } & \cdots & {F_{a\left( k \right)} } & {F_{b\left( k \right)} } & \cdots & {F_{{a\left( {\eta N_{\max } } \right)}} } & {F_{{b\left( {\eta N_{\max } } \right)}} } \\ \end{array} } \right]^{{\text{T}}} . $$

The relevant variables used are as follows: $\varpi t = \psi$ and $\varpi = \frac{\omega }{{\omega_{n} }}$, the non-dimensionalized time scale and normalized frequency value with the natural frequency $\omega_{n}$; $T = \eta \tau$, the time period corresponding to $0 \le t < T$ → $0 \le \psi < \frac{2\pi }{{\omega_{n} }}$; $\eta$, a sub-harmonic index; $\tau$, a fundamental excitation frequency; *k*, an incremental index $k = \omega_{n} , 2\omega_{n} , 3\omega_{n} \cdots$. By employing the relationships of $\dot{\theta }\left( t \right) = \frac{d\theta }{{dt}} = \varpi \frac{d\theta }{{d\psi }} = \varpi \theta^{\prime}$ and $\mathop \theta \limits \left( t \right) = \varpi^{2} \theta^{\prime\prime}$ in Eq. (7), the overall Galerkin scheme of the basic equation is defined as follows.

10a,b $$ \underline{{{\underline{\mathbf {H} }}}} \underline{{{\underline{\mathbf {\Psi } }}}} = \underline {0} ,\quad {\underline{\mathbf {\Psi } }} = - \varpi^{2} m\underline{{{\underline{\mathbf {P} }}}}^{^{\prime\prime}} \underline{{{{\varvec{\uptheta}}}_{{\mathbf{c}}} }} + \varpi c\underline{{{\underline{\mathbf {P} }}}}^{{\prime}} \underline{{{{\varvec{\uptheta}}}_{{\mathbf{c}}} }} + \underline{{{\mathbf{f}}_{{{\mathbf{nc}}}} }} - \underline{{{\mathbf{F}}_{{{\mathbf{Ec}}}} }} = \underline {0} . $$

To calculate the solutions for $\underline{{{{\varvec{\uptheta}}}_{{\mathbf{c}}} }}$ in Eq. (10) with their relevant parameter $\varpi$, the Newton–Raphson method is used based on the condition ${\underline{\mathbf {\Psi } }} \to \underline {0}$, where ${\underline{\mathbf {\Psi } }}$ is considered as a function of $\underline{{{{\varvec{\uptheta}}}_{{\mathbf{c}}} }}$ and $\varpi$, i.e., ${\underline{\mathbf {\Psi } }}\left( {\underline{{{{\varvec{\uptheta}}}_{{\mathbf{c}}} }} ,\varpi } \right)$. Using the Newton–Raphson method, the solutions for $\underline{{{{\varvec{\uptheta}}}_{{\mathbf{c}}} }}$ and $\varpi$ for each step were determined, as presented in previous publications^16,17^.

### Examination of initial results according to frequency upsweeping

**Figure 2:**
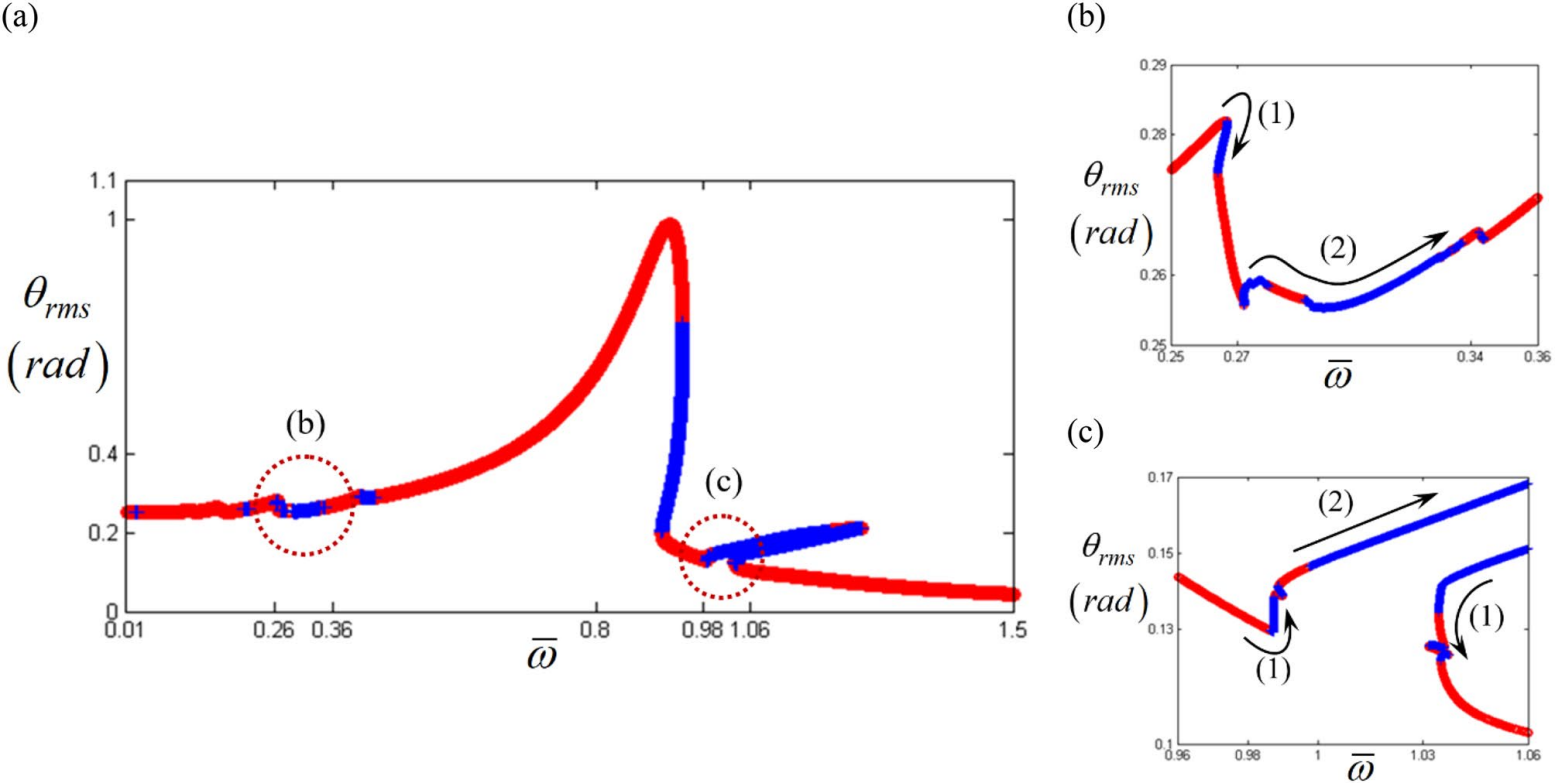
Nonlinear frequency response with RMS reflecting the stability conditions under the frequency upsweeping: (**a**) RMS values with super- and sub-harmonic regimes; (**b**) super-harmonic regimes and their dynamic flows; (**c**) sub-harmonic regimes and their dynamic flows. Key: circle, stable solutions; plus, unstable solutions.

Figure 2 shows the initial results obtained by the HBM with $\eta$ = 2 and *N*_max_ = 24 under the frequency upsweeping condition. In order to capture the nonlinear responses induced by the asymmetrical piecewise type nonlinearities, sufficient number such as *N*_max_ = 24 is employed based on the prior studies^16,17,28^. Also, this study focuses on frequency upsweeping conditions to examine the nonlinear dynamic characteristics. To examine the stability conditions, Hill’s method was employed in this study^16,17,27^. Here, the red dotted lines marked with (b) and (c) in Fig. 2a indicate the super- and sub-harmonic response regimes. To obtain the sub-harmonic responses, a small range of excitation input values is employed artificially^10^. For this study, the valid components of the input torque vector with $\eta$ = 2 and *N*_max_ = 24 are expressed as $F_{m} = 168.9$, $F_{a\left( 2 \right)} = - 87.97$ and $F_{b\left( 2 \right)} = 235.65$ in Eq. (9). To trigger the sub-harmonic responses, the components of the input torque pertaining to the sub-harmonic locations, such as $F_{a\left( 1 \right)}$ and $F_{b\left( 1 \right)}$, are expressed as $F_{a\left( 1 \right)} = \varepsilon F_{a\left( 2 \right)}$ and $F_{b\left( 1 \right)} = \varepsilon F_{b\left( 2 \right)}$, respectively. Here, the value employed for $\varepsilon$ is $1 \times 10^{ - 5}$. Figure 2b,c) show the system responses in the super- and sub-harmonic regimes, respectively, where the red circles and blue cross lines indicate stable and unstable conditions, respectively. In these regimes, the stability conditions clearly show variation, such as from stable to unstable (STU) and from unstable to stable (UTS) along the arc length under the frequency upsweeping condition. For example, the locations marked with (1) in Fig. 2b,c illustrate that the direction of the solutions changes abruptly from upsweeping to reverse (or from reverse to upsweeping) with the STU (or the UTS). However, the location marked with (2) indicates that the direction of the solutions follows the same trend as the frequency upsweeping with the STU (or the UTS). Thus, as suggested in the specific objectives with regard to the direction of arc length and their relevant stability conditions, this study investigated the nonlinear dynamic characteristics by projecting their responses in time and FFT domains, phase planes, and Poincare maps and compared them with bifurcation diagrams.

### Examination of super-harmonic responses

**Figure 3:**
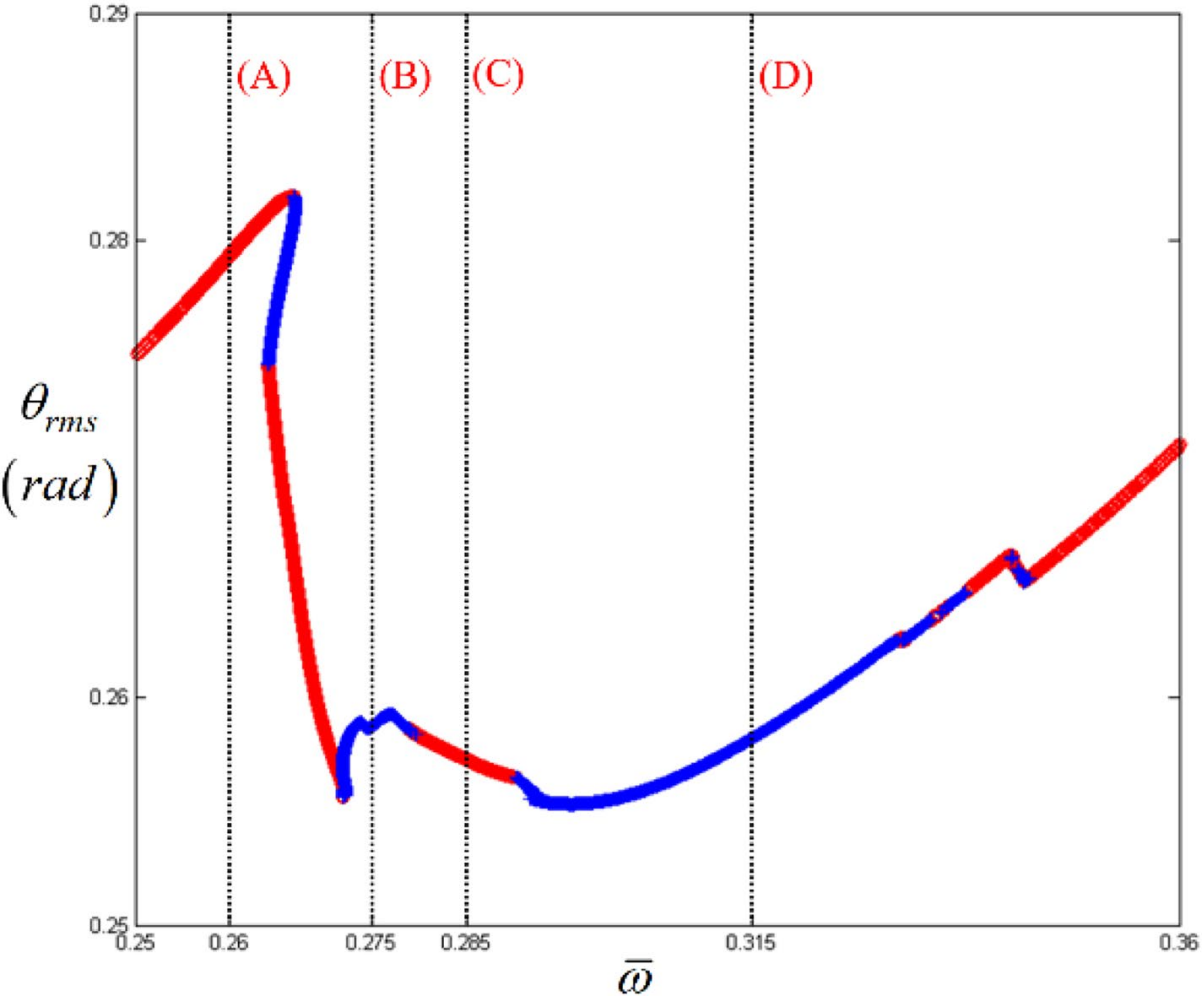
Nonlinear frequency responses focused on super-harmonic regimes with RMS values. Key: circle, stable solutions; plus, unstable solutions.

**Figure 4:**
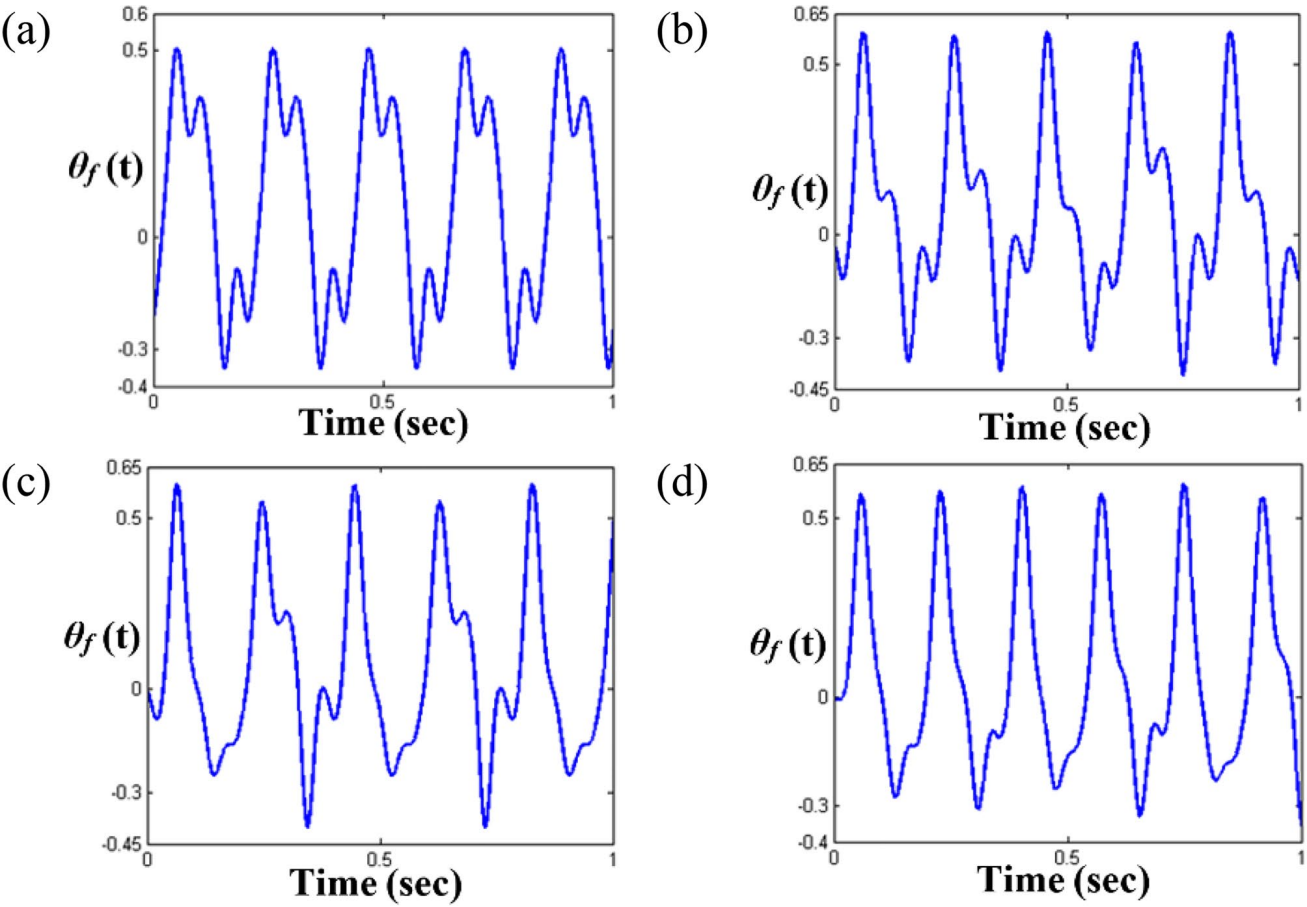
Comparisons of time histories calculated by NS in the super-harmonic regimes: (**a**) time history at $\varpi = 0.26$; (**b**) time history at $\varpi = 0.275$; (c) time history at $\varpi = 0.285$; (d) time history at $\varpi = 0.315$.

**Figure 5:**
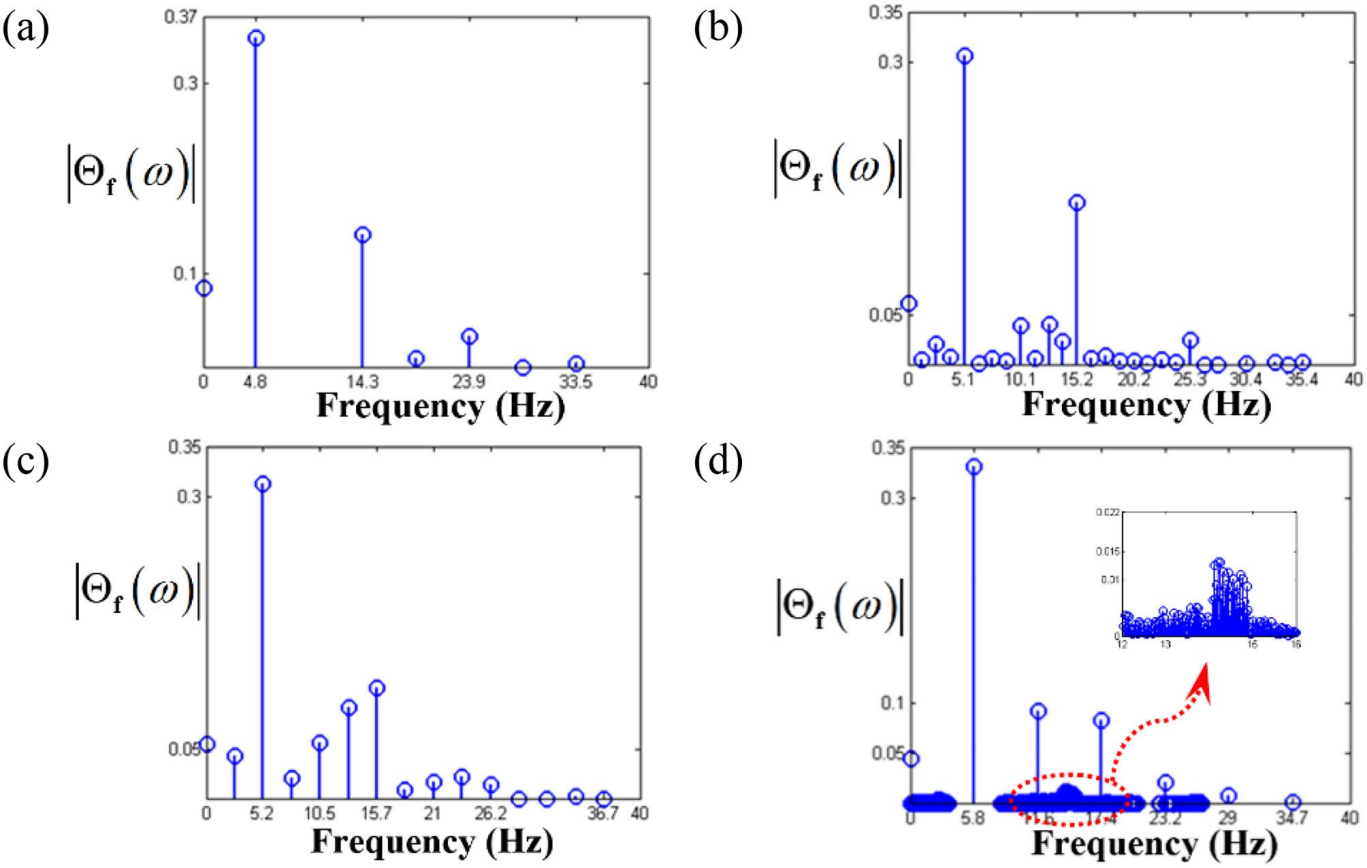
Comparisons of FFT results based on 200 cycles of time histories: (**a**) FFT results at $\varpi = 0.26$; (**b**) FFT results at $\varpi = 0.275$; (c) FFT results at $\varpi = 0.285$; (d) FFT results at $\varpi = 0.315$.

**Figure 6:**
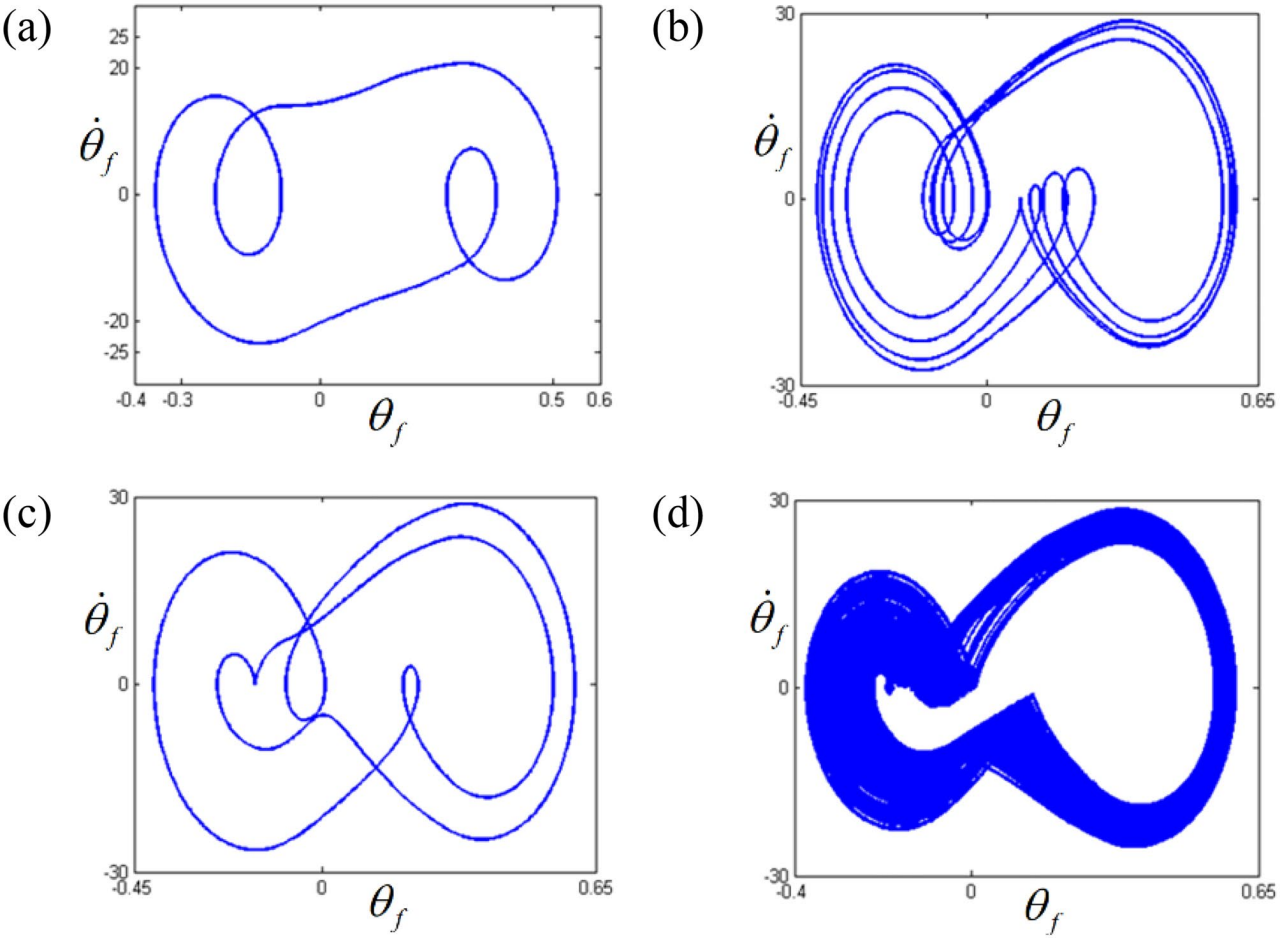
Comparisons of phase diagrams according to different frequency regimes: (**a**) phase diagram at $\varpi = 0.26$; (**b**) phase diagram at $\varpi = 0.275$; (**c**) phase diagram at $\varpi = 0.285$; (**d**) phase diagram at $\varpi = 0.315$.

**Figure 7:**
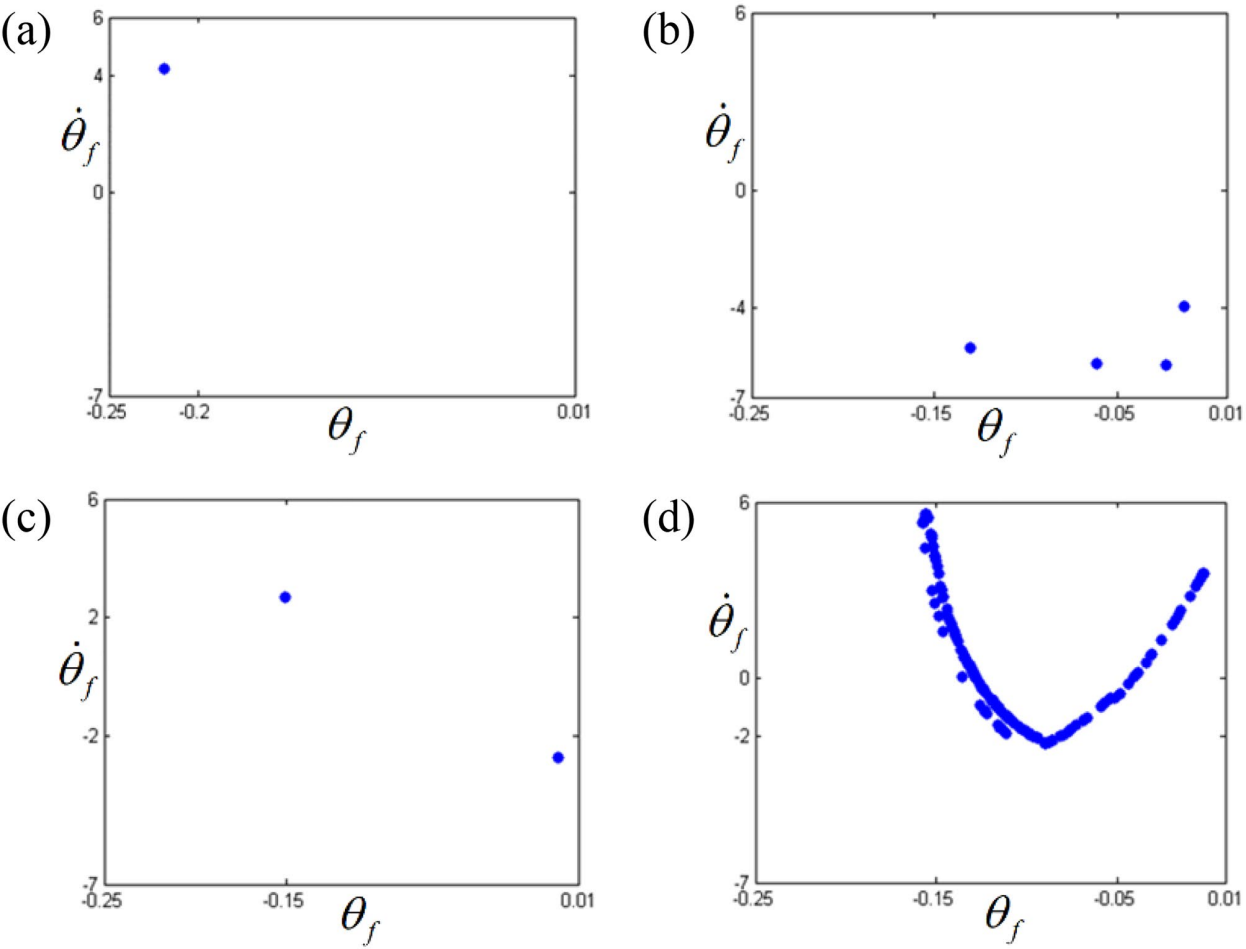
Comparisons of Poincare maps according to different frequency regimes: (**a**) Poincare map at $\varpi = 0.26$; (**b**) Poincare map at $\varpi = 0.275$; (**c**) Poincare map at $\varpi = 0.285$; (d) Poincare map at $\varpi = 0.315$.

Figure 3 shows a detailed view of the super-harmonic responses presented in Fig. 2. To examine the nonlinear dynamic behaviors, their system responses can be analyzed along the various regimes indicated by (A), (B), (C), and (D), where the normalized frequencies are 0.26, 0.275, 0.258, and 0.315, respectively. Here, the STU regime is located between (A) and (B), where the direction of the arc length is observed to reverse. In general, this point is related to the saddle-node point where the jumping phenomenon occurs; thus, numerical or experimental results cannot be obtained. Therefore, this study focuses on investigating the nonlinear responses in the same direction as the frequency upsweeping, where the STU and UTS are changed, because, in previous studies, the dynamic behaviors estimated as unstable conditions have not been investigated thoroughly with respect to the practical motions of the system^7^. In Fig. 3, (A) indicates the resonance at the super-harmonic regimes under the stable condition. (C) shows another stable condition around the super-harmonic regime with the same direction as the frequency upsweeping; however, it is located between two unstable regimes marked with (B) and (D). Here, two unstable regimes, (B) and (D), are found with the same arc-length direction as the frequency upsweeping. Figs. 4, 5, 6 and 7 show the simulation results with time histories, FFT results, phase diagrams, and Poincare maps according to regimes (A), (B), (C), and (D).

Figure 4 compares the time histories in regimes (A), (B), (C), and (D). Figure 4a clearly indicates the super-harmonic response, which is also observed in the FFT results, as shown in Fig. 5a, because the nonlinear response is located in the stable regimes, as shown in Fig. 3. As the frequency range changes to that of regimes (B), (C), and (D), their dynamic behaviors become complex, as evident from the time histories shown in Fig. 4. When the responses of regimes (B), (C), and (D) are examined carefully, it is observed that the dynamic characteristics are closely related to the stability conditions. For example, when the time histories of regimes (C) and (B) (or (D)) are compared with each other, the dynamic behavior in regime (C) clearly includes the number of harmonic components dependent on the fundamental frequency rather than the ones in regime (B) (or (D)). When the dynamic response of regime (C) is considered with respect to the stability conditions, it reflects the stable condition. However, the dynamic responses in regimes (B) and (D) were estimated as unstable conditions.

In addition, the FFT results in Fig. 5 indicate the relevance of the dynamic characteristics corresponding to the stability conditions. For instance, Fig. 5c shows that the system responses consist of harmonic terms relevant to their fundamental harmonic components such as *ω* = 5.2 Hz (or $\varpi$ = 0.285). However, the system responses in the unstable regimes (B) and (D) reflect various numbers of harmonic components, as shown in Fig. 5b,d. The dynamic behavior in regime (D), shown in Fig. 5d, exhibits the presence of the most complex components.

The dynamic behaviors under various stability conditions with respect to the STU and UTS can be thoroughly examined when their responses are projected in phase diagrams and Poincare maps, as shown in Figs. 6 and 7. To determine the phase diagrams and Poincare maps using the NS, this study employed the modified Runge-Kutta method^29^. The system responses were estimated for 500 cycles until the transient response effects were completely removed, and the last 200 cycles were considered to obtain the results. The system response in regime (A), shown in Fig. 6a, clearly indicates three dominant circles, which consist of only one dot in the Poincare map, as shown in Fig. 7a. Compared with the dynamic behavior in regime (A), the other motions in regimes (B), (C), and (D) show more complex circles. When the dynamic behavior in the stable regime (C) is examined, it is evident that the phase diagram is also composed of a finite number of circles, and the number of dots in the Poincare map is relatively low, e.g., two dots, as shown in Fig. 7c. However, the dynamic responses in the unstable regimes (B) and (D) show a greater number of circles, even though the overall shape of the circle is evident from Fig. 6b, d. These dynamic characteristics are projected well in the Poincare maps, as shown in Fig. 7b, d. In particular, the dynamic behavior in regime (D) shows a chaotic phenomenon, which is reflected by various numbers of dotted lines, as shown in Fig. 7d.

Based on the examination of the dynamic responses along different frequency regimes by considering the stability conditions, their dynamic behaviors can be understood more clearly when they are projected in bifurcation diagrams. To create the bifurcation diagrams, 200 cycles of RMS values were obtained from each cycle.

**Figure 8:**
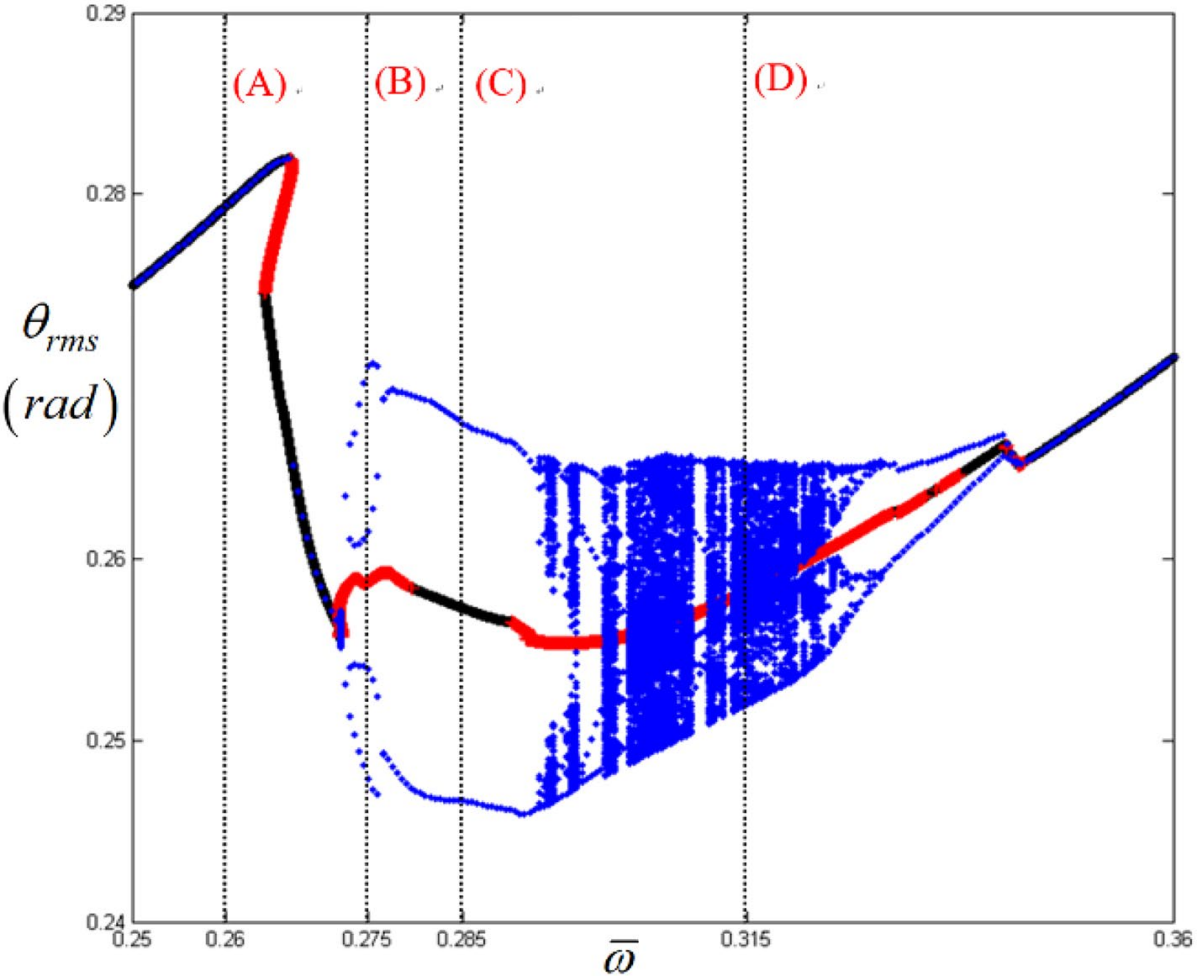
Comparison of HBM and bifurcation diagram with RMS values focused on super-harmonic response regime. Key: dashed line, HBM result with stable solutions; plus**,** HBM result with unstable solutions; circle, bifurcation diagrams.

Overall, when the dynamic behaviors in regimes (A), (B), (C), and (D), shown in Figs. 4, 5, 6 and 7, are compared with the bifurcation diagram shown in Fig. 8, the response in each regime can be determined in a clear way. For example, regime (A) does not show any bifurcation, which has already been confirmed as a pure super-harmonic response. As the response transitions into regimes (B), (C), and (D), the relevant bifurcation of these regions is clearly observed with complex characteristics. When the stability conditions are compared with each bifurcation status, regime (B) corresponding to the unstable condition shows a period-doubling cascade immediately after the period-doubling located between regimes (A) and (B). Regime (C) returns to the period-doubling status with the stable condition. When the regime is changed to (D) under the STU condition, the bifurcation becomes more complicated and reflects the chaotic response. Table 3 summarizes the dynamic characteristics under various conditions based on the stability and arc-length direction. In the table, the symbols “+” and “−” indicate the same and reverse directions of frequency upsweeping.

**Table 3:** Dynamic characteristics in the super- and sub-harmonic regimes based on frequency upsweeping.

Stability conditions	Sweeping direction	Dynamic characteristics
Stable → Unstable (STU)	+ → −	Saddle-node point
	+ → +	Period-doubling cascade
Unstable → Stable (UTS)	+ → −	Period-doubling
	+ → +	

## Results of sub-harmonic responses

**Figure 9:**
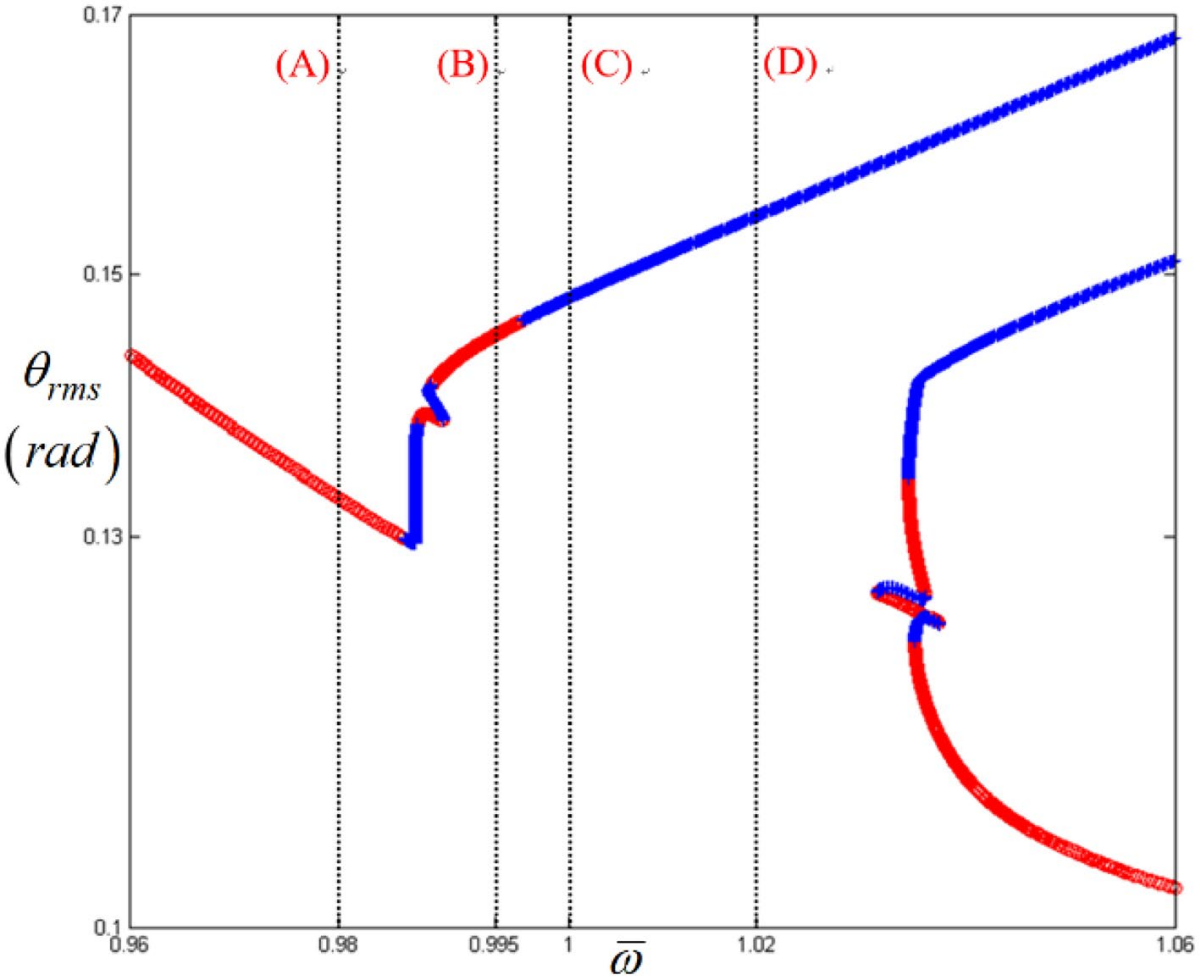
Nonlinear frequency responses focused on sub-harmonic regimes with RMS values. Key: circle, stable solutions; plus, unstable solutions.

Figure 9 presents a detailed view of the sub-harmonic responses shown in Fig. 2. The system responses can be analyzed along various regimes indicated by (A), (B), (C), and (D), where the normalized frequencies $\varpi$ are 0.98, 0.995, 1.0, and 1.02, respectively. Here, the STU regime is found between (A) and (B), where the direction of arc length is reversed. In Fig. 9, regime (A) shows the fundamental harmonic response area immediately before the sub-harmonic responses occur.

**Figure 10:**
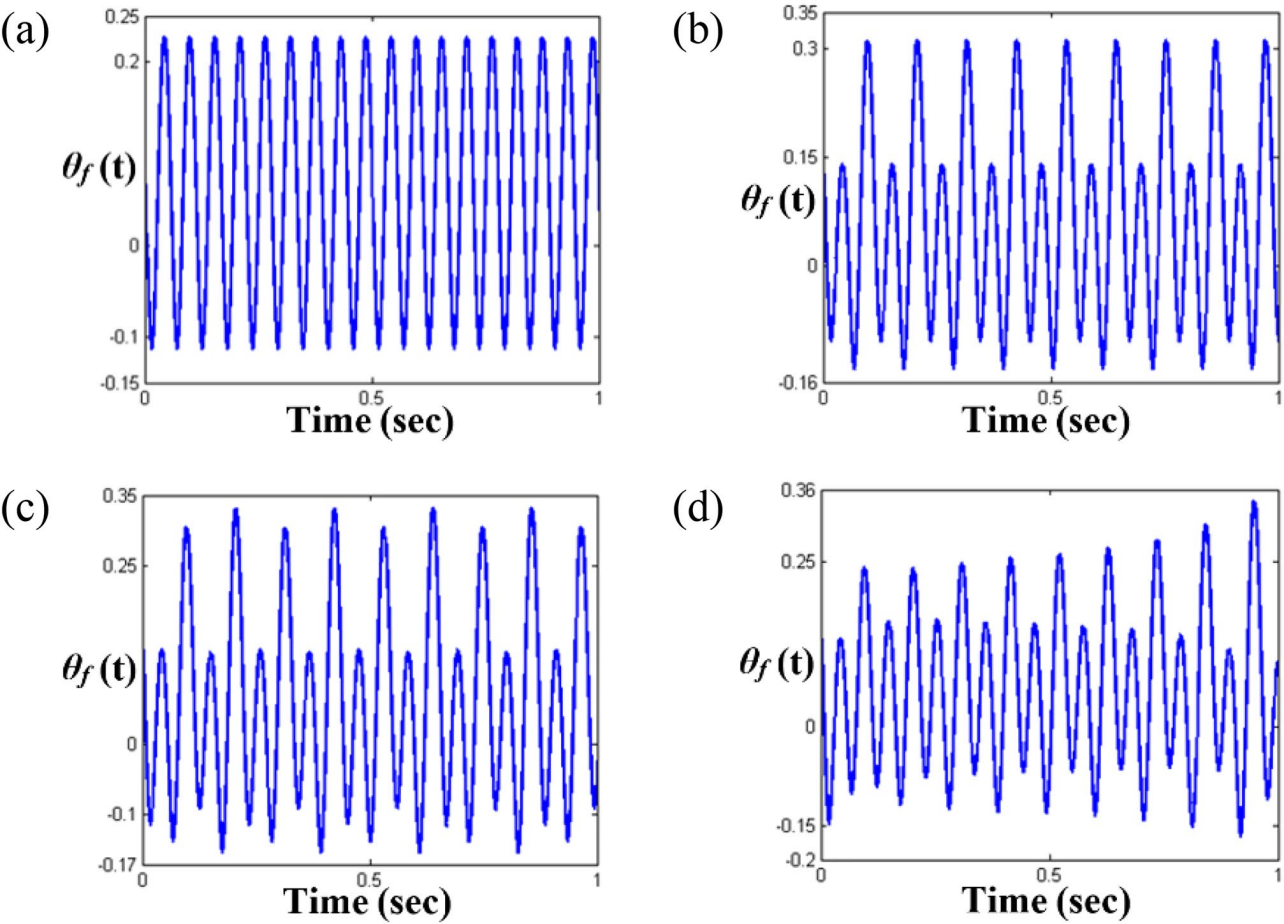
Comparisons of time histories calculated by NS in the sub-harmonic regimes: (**a**) time history at $\varpi = 0.98$; (**b**) time history at $\varpi = 0.995$; (**c**) time history at $\varpi = 1.0$; (**d**) time history at $\varpi = 1.02$.

**Figure 11:**
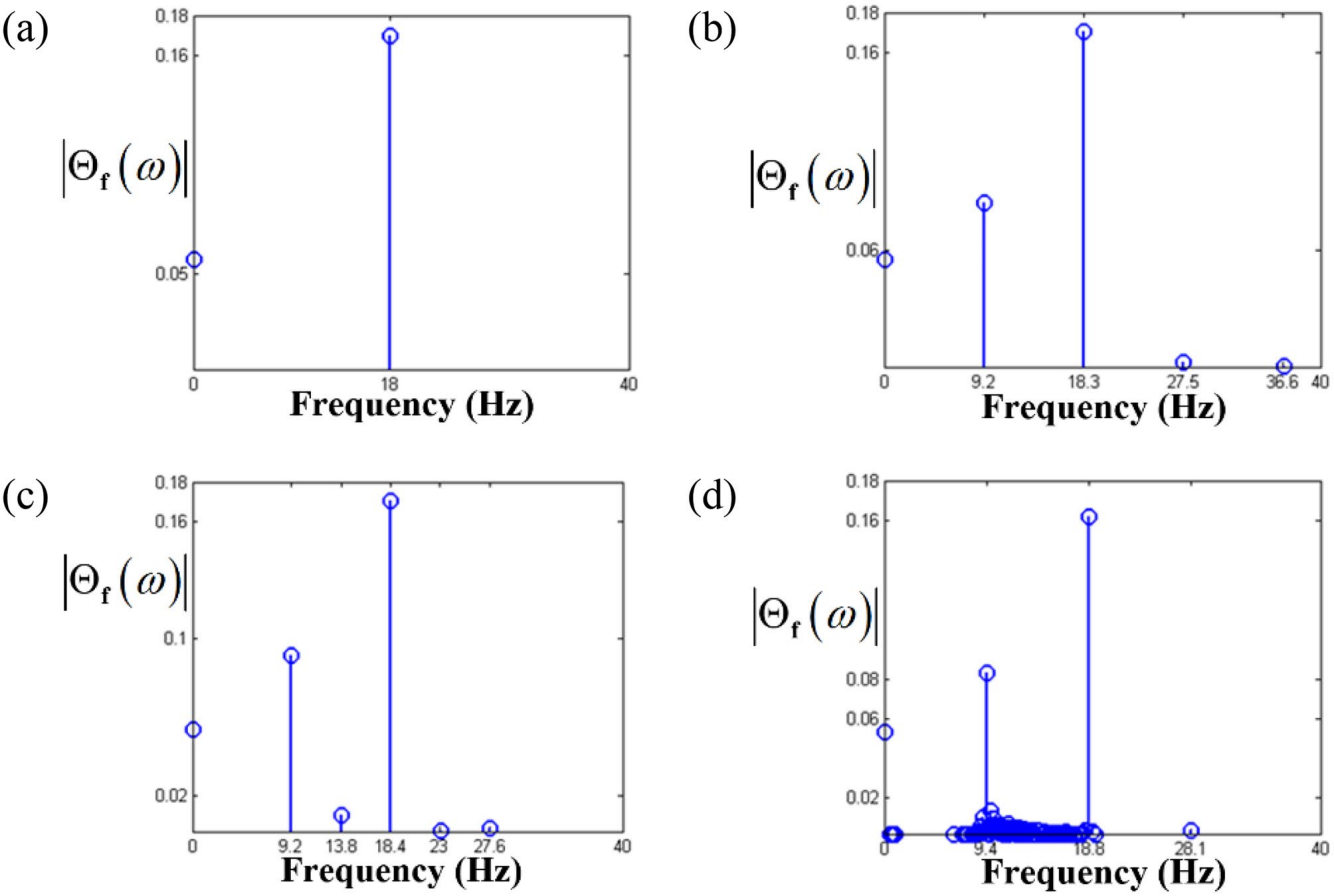
Comparisons of FFT results based on 200 cycles of time histories: (**a**) FFT results at $\varpi = 0.98$; (**b**) FFT results at $\varpi = 0.995$; (**c**) FFT results at $\varpi = 1.0$; (d) FFT results at $\varpi = 1.02$.

These are clearly observed in Figs. 10 and 11, respectively. Among the sub-harmonic response areas, i.e., regimes (B), (C), and (D), regimes (C) and (D) pertain to the unstable condition. The arc length in the unstable regimes (C) and (D) follows the same direction as the frequency upsweeping. As the frequency regime transitions from (A) to (B), the dynamic behaviors clearly reflect the sub-harmonic effects in regime (B). When the time histories and FFT results shown in Figs. 10a,b and 11a,b are compared, the variation in the dynamic characteristics is easily confirmed. As the frequency ranges changes to that of regimes (C) and (D), the dynamic behaviors become more complex than those of regimes (A) and (B), as shown in Figs. 10c,d and 11c,d. When the dynamic responses of regimes (C) and (D) are examined, it is evident that the dynamic characteristics are closely related to the stability conditions, which was revealed previously in the super-harmonic area. The dynamic behaviors in regimes (C) and (D) show that they contain more harmonic components, as indicated in Figs. 10c,d and 11c,d. In particular, the dynamic response in regime (D) includes various harmonic components, as shown in Fig. 11d.

**Figure 12:**
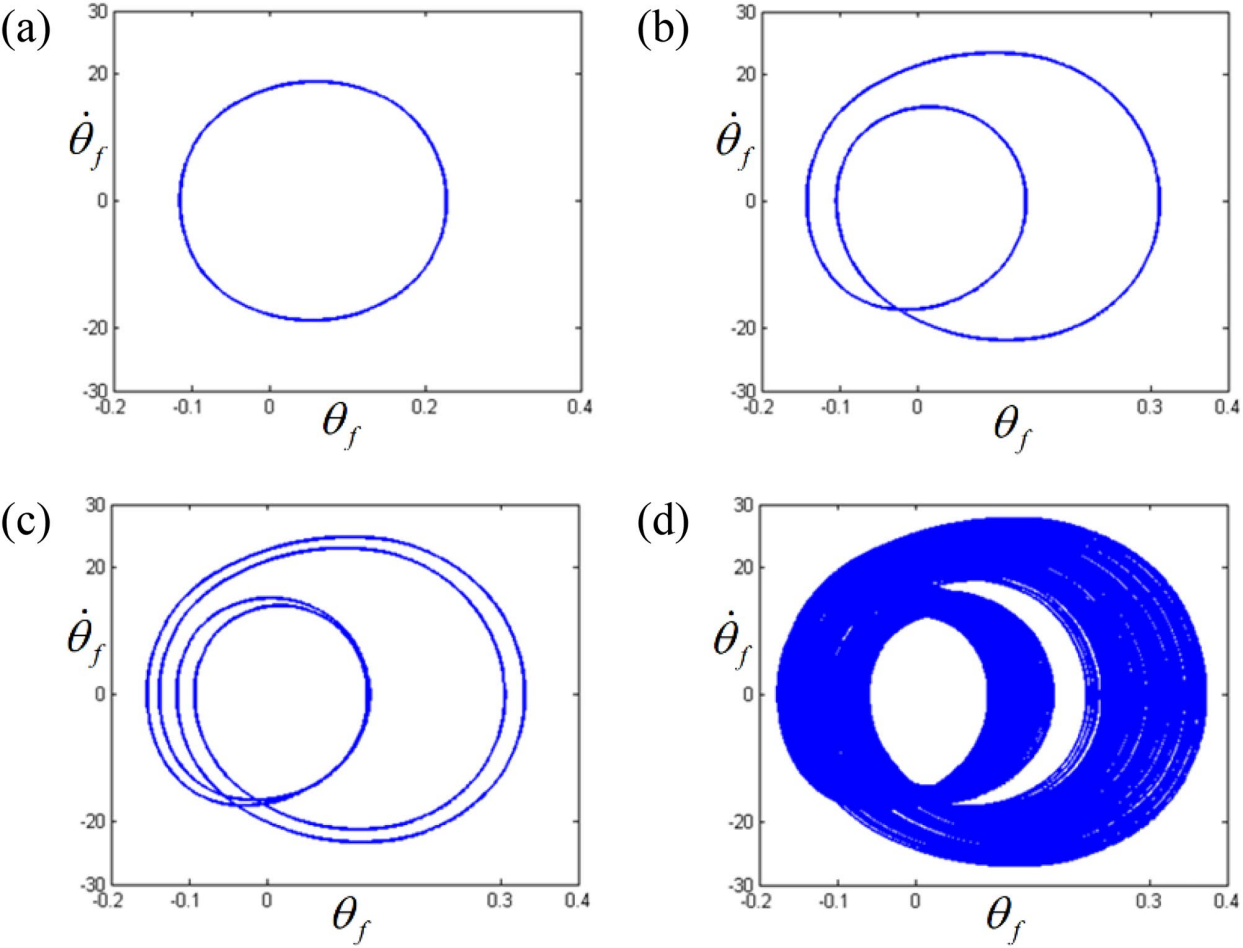
Comparisons of phase diagrams according to different frequency regimes: (**a**) phase diagram at $\varpi = 0.98$; (**b**) phase diagram at $\varpi = 0.995$; (**c**) phase diagram at $\varpi = 1.0$; (**d**) phase diagram at $\varpi = 1.02$.

**Figure 13:**
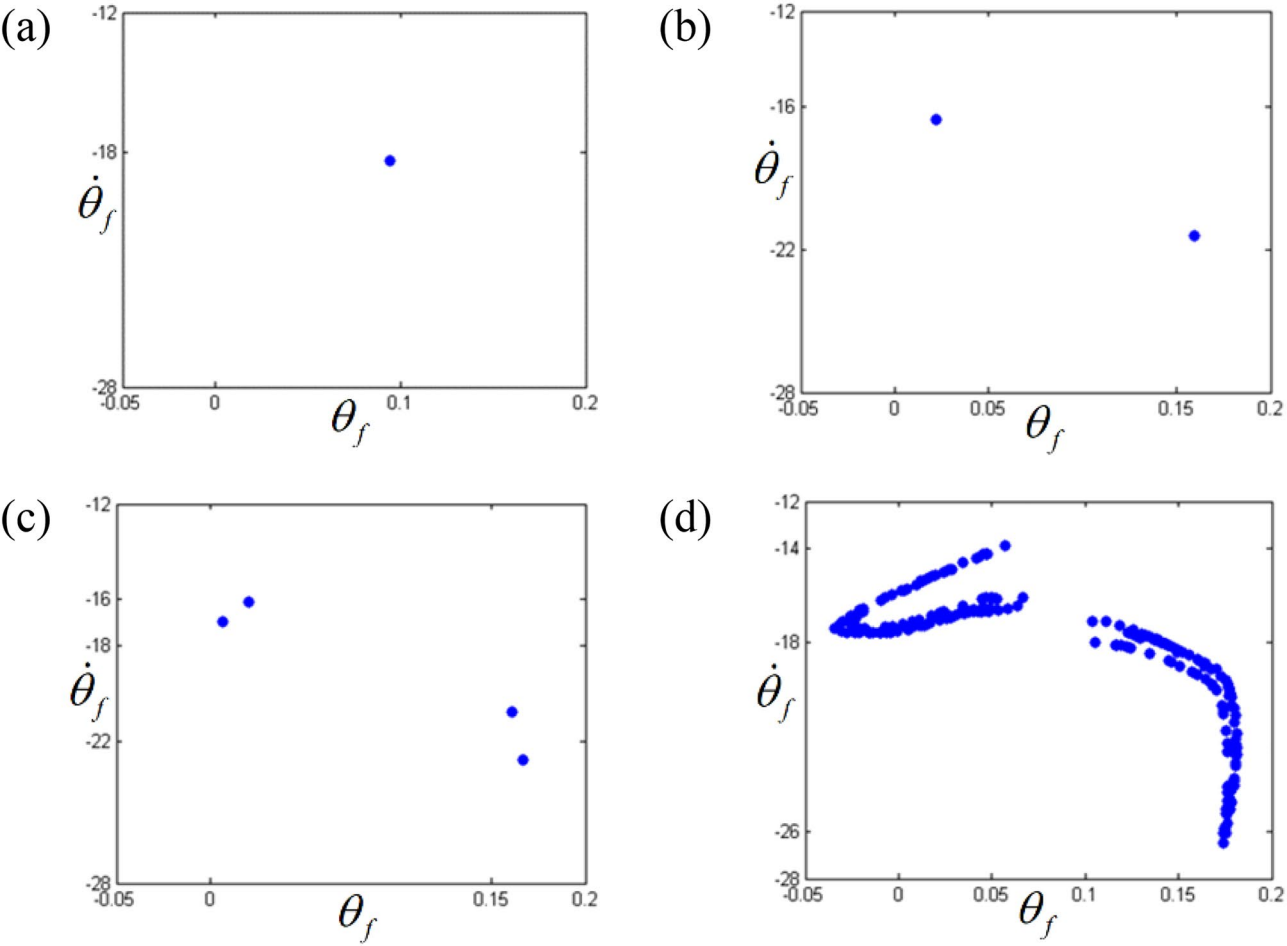
Comparisons of Poincare maps according to different frequency regimes: (**a**) Poincare map at $\varpi = 0.98$; (**b**) Poincare map at $\varpi = 0.995$; (**c**) Poincare map at $\varpi = 1.0$; (d) Poincare map at $\varpi = 1.02$.

The dynamic behaviors in the sub-harmonic area with respect to STU and UTS were also analyzed using phase diagrams and Poincare maps, as shown in Figs. 12 and 13. From the phase diagrams, the number of circles increases as the frequency range transitions from regime (A) to regime (D). In particular, regime (D) shows a large number of periodic circles, where chaotic motions are expected. These variational dynamic conditions were examined based on the Poincare maps, as shown in Fig. 13. As the regimes transition from (A) to (D), the number of periodic points increases. Figure 13d shows the chaotic motions clearly as variational periodic points are passed through.

**Figure 14:**
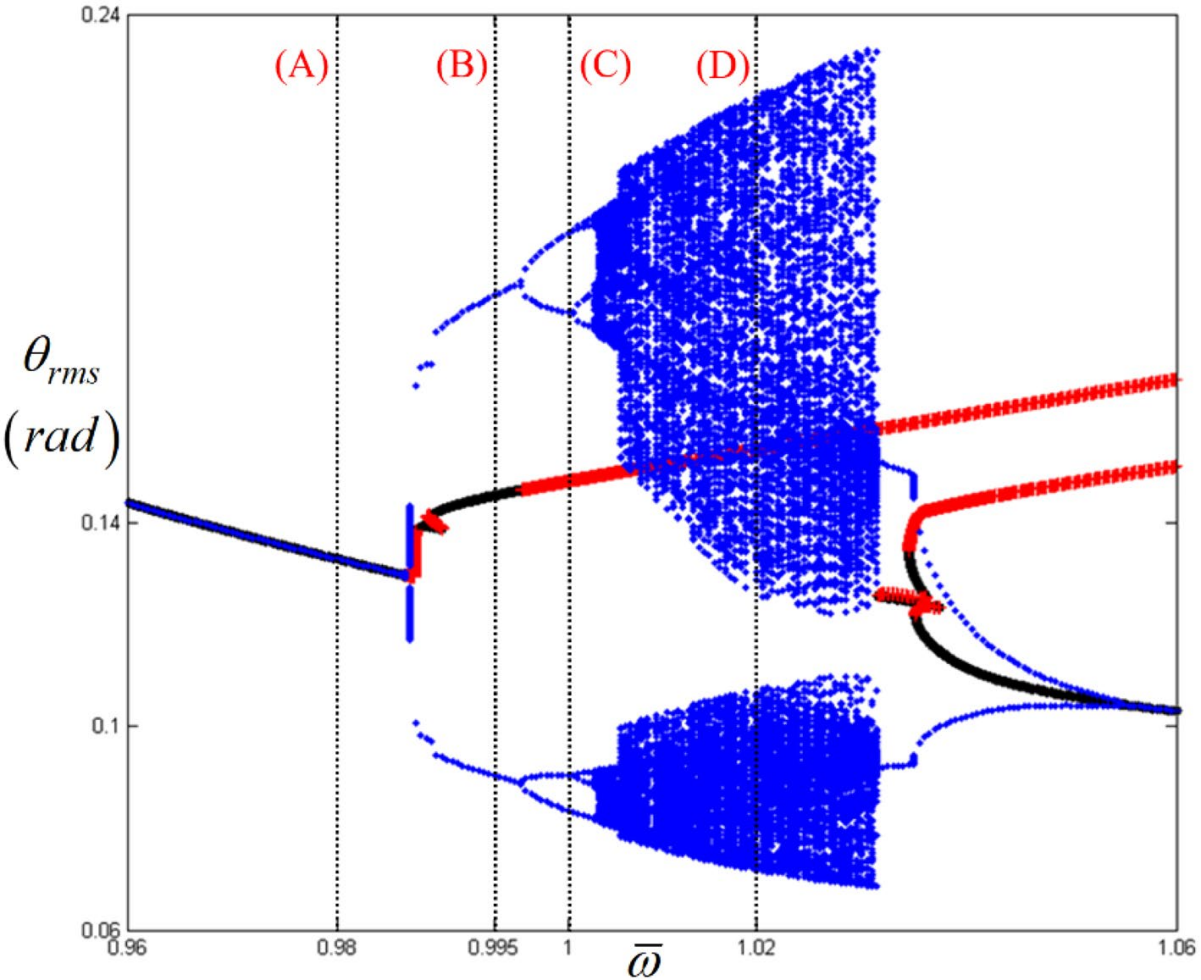
Comparison of HBM and bifurcation diagram with RMS values focused on sub-harmonic response regime. Key: dashed line, HBM result with stable solutions; plus**,** HBM result with unstable solutions; circle, bifurcation diagrams.

Overall, when the dynamic behaviors in regimes (A), (B), (C), and (D) in Figs. 10, 11, 12 and 13 are compared in a bifurcation diagram, as shown in Fig. 14, the strong relationship between the dynamic behaviors and stability conditions is evident, which was examined previously for the super-harmonic area. For example, regime (A) only shows the fundamental harmonic response. However, bifurcation is revealed when the frequency regime transitions into (B), (C), and (D). When the system responses are stable in regime (B), as evident from Fig. 14, they exhibit period-doubling, which is related to the sub-harmonic responses. However, when the system responses fall into unstable conditions such as regimes (C) and (D), as indicated in Fig. 14, the dynamic behaviors show the period-doubling cascade and finally become chaotic. This is also well correlated, as shown in Table 3.

## Conclusion

This study analyzed the nonlinear dynamic characteristics of super- and sub-harmonic response areas. To understand the nonlinear motions related to the unstable regimes, this study suggests the use of stability and bifurcation analysis. First, the dynamic characteristics in the super- and sub-harmonic areas according to the variations in stability conditions were investigated using the HBM. Here, the stability variations along the direction of the arc length were proved to have a close relationship with nonlinear dynamic behaviors such as period-doubling, period-doubling cascade, and chaotic motions. Second, this study analyzed the nonlinear dynamic responses by comparing the bifurcations with the stability conditions. To understand the variation of dynamic behaviors, FFT results, phase diagrams, and Poincare maps were also compared.

Furthermore, to investigate and understand the nonlinear dynamic behaviors under the STU and UTS conditions by projecting them in bifurcation diagrams, this study determined the relevant arc-length direction empirically based on the NS results. However, the method to verify the dynamic characteristics with respect to the arc-length direction and stability conditions can be defined analytically, which will be the future scope of this work.
